# Neuronal Ceroid Lipofuscinosis-like Disorder in a Dachshund with Sequence Variants in Lysosome-Related Genes

**DOI:** 10.3390/genes17040465

**Published:** 2026-04-15

**Authors:** Joan R. Coates, Kristen Keyes, Rebecca E. H. Whiting, Juri Kuroki, Brandie Morgan-Jack, Tendai Mhlanga-Mutangadura, Keiichi Kuroki, Martin L. Katz

**Affiliations:** 1 Department of Veterinary Medicine and Surgery, College of Veterinary Medicine, University of Missouri, Columbia, MO 65211, USA; coatesj@missouri.edu (J.R.C.); k.keyes@missouri.edu (K.K.); otaj@missouri.edu (J.K.); 2 Department of Ophthalmology, School of Medicine, University of Missouri, Columbia, MO 65212, USA; whitingre@health.missouri.edu (R.E.H.W.); morganbr@health.missouri.edu (B.M.-J.); 3 Department of Pathobiology and Integrative Biomedical Sciences, College of Veterinary Medicine, University of Missouri, Columbia, MO 65211, USA; tendai@missouri.edu (T.M.-M.); kurokik@missouri.edu (K.K.)

**Keywords:** lysosomal storage disease, lipofuscin, neurodegeneration, whole genome sequencing, autophagy, canine

## Abstract

Background/Objectives: Among the most common hereditary neurodegenerative disorders in people are the neuronal ceroid lipofuscinoses (NCLs), a subgroup of lysosomal storage disorders. For most cases of NCL, the genes containing the causative variants have been identified. NCLs also occur in dogs, and in most instances variants responsible for the canine NCLs occur in genes orthologous to those associated with the human disorders. An adult miniature Dachshund presented with clinical signs consistent with NCL. Studies were undertaken to determine whether the disease phenotype supported the classification of the disease as an NCL and to identify potential causal DNA sequence variants. Methods: The proband underwent complete neurological and ophthalmological examinations followed by euthanasia. Tissues were examined for NCL-like pathology. Whole genome sequence analysis (WGS) was performed. Results: The clinical signs and tissue pathology were consistent with those of NCL disease, although with some features distinct from previously described forms of canine NCL. The proband was uniquely homozygous for variants in five genes associated with lysosomal function, four of which have not previously been associated with the NCLs. Conclusions: The proband suffered from a novel NCL-like disorder. Determining whether one or a combination of more than one of the five potentially causal DNA sequence variants was responsible for the disease will require evaluation of additional cases.

## 1. Introduction

Among the most common progressive hereditary neurodegenerative disorders in people are the neuronal ceroid lipofuscinoses (NCLs), of which there are multiple forms [1,2]. There is substantial variability in disease signs within this group of disorders, but all forms are characterized by a base set of neurological signs. These include brain atrophy and progressive loss of motor and cognitive skills. Other common features include loss of vision due to retinal degeneration and seizures. A unifying histopathological feature of the NCLs is the accumulation of autofluorescent lysosomal storage bodies in neurons and other cell types. Most forms of NCL are ultimately fatal.

Historically, the human NCLs were classified based on clinical signs, including age of onset, rate of disease progression, and patterns of clinical signs. However, with advances in molecular genetics, the genetic variants that underlie most forms of NCL have now been identified, and the diseases are now classified based on the gene that contains the causal variant. There are currently 13 genes that are widely recognized as containing variants associated with a form of NCL [2,3]. However, variants in a number of additional genes have also been associated with NCL-like disorders [4,5,6,7,8,9,10,11,12,13,14,15].

Naturally occurring NCLs have been identified in species that include mice, sheep, monkeys, cats, and dogs [16,17,18,19,20,21,22,23,24]. Among dogs presenting with NCL-like signs, at least 23 causal variants in 12 genes have been identified [4,5,6,7,24,25,26,27,28,29,30,31,32,33,34,35,36,37,38,39,40,41]. Dogs exhibiting signs consistent with NCL can now be easily screened for these known variants. Many cases still occur in which a dog presents with NCL-like signs but does not have any of the known causal variants. In these cases, whole genome sequencing (WGS) can be used to screen for potential disease variants.

In this study, a miniature Dachshund that presented with a late-onset disorder suggestive of NCL was evaluated. Analyses were performed to characterize the disease phenotype and WGS was performed to identify potential causal DNA sequence variants.

## 2. Materials and Methods

### 2.1. History and Physical and Neurological Examination

A 6 year and 3-month-old male neutered dapple miniature Dachshund was presented to the University of Missouri Veterinary Health Center (MU VHC) for suspected signs of recurrence of hepatic encephalopathy. A thorough history was provided by the pet owner. A complete physical examination was performed on the dog. A neurological examination was performed by a board-certified veterinary neurologist and resident in training. The neurological examination consisted of observation of mentation, posture and gait, cranial nerve evaluation, postural reaction testing, spinal reflexes, spinal palpation and pain assessment. The pet owner consented to additional diagnostic procedures.

### 2.2. Magnetic Resonance Imaging of the Brain

Magnetic resonance imaging (MRI; Canon Vantage Titan 3T MRI system, Canon Medical Systems USA, Tustin, CA, USA) of the brain was performed with the dog under general anesthesia. The dog was sedated with intravenously administered 0.002 mg/kg dexmedetomidine HCl (Dexmedesed, Dechra Veterinary Products, Overland Park, KS, USA) and 0.25 mg/kg methadone HCl, USP (Long Grove Pharmaceuticals, LLC, Rosemont, IL, USA). Anesthesia was induced with intravenously administered 4 mg/kg to effect propofol injection emulsion (Avet Pharmaceuticals Inc. East Brunswick, NJ, USA) and maintained by inhalation sevoflurane (Sevospire, Dechra Veterinary Products, Overland Park, KS, USA) at a minimum alveolar concentration between 1 and 2. The dog was positioned in dorsal recumbency for imaging. Sequences obtained included T2-weighted (T2W) images, and T1-weighted (T1W) (pre- and post-contrast; Omniscan IV) in sagittal, transverse, and dorsal planes. In addition, T2-weighted fluid attenuated inversion recovery (FLAIR), diffusion weighted and T2 * gradient echo (GRE) images in transverse planes were acquired.

### 2.3. Ophthalmologic Examination

A complete ophthalmic examination was performed by a board-certified veterinary ophthalmologist at 6 years 3 months of age, including Schirmer tear test-1 (Merck Animal Health, Summit, NJ, USA), Tonovet tonometry (TonoVet^®^; Icare Finland Oy, Vantaa, Finland), corneal application of fluorescein stain (Bio-Glo; HUB Pharmaceuticals, LLC, Scottsdale, AZ, USA), slit-lamp biomicroscopy (Kowa SL-17; Kowa Co., Tokyo, Japan), and indirect ophthalmoscopy with a binocular headset and a 28D condensing lens (Keeler Vantage; Keeler Instruments, Inc., Mavern, PA, USA and Volk Optical Inc., Mentor, OH, USA, respectively). The pupils were dilated with topical tropicamide 1% ophthalmic solution USP (Akorn, Lake Forest, IL, USA) for fundic evaluation and imaging. Fundus photos were acquired using a handheld retinal camera (Optomed Aurora; Optomed, Oulu, Finland). Optical coherence tomography (OCT) and scanning laser ophthalmoscopy (SLO) images of the retina were captured with a Heidelberg Spectralis HRA/OCT (Heidelberg, Germany) while the dog was lightly sedated with dexmedetomidine (6 mcg/kg) (Parenell Pharmaceuticals, San Rafael, CA, USA) and midazolam (0.25 mg/kg) (Pfizer, New York, NY, USA).

### 2.4. Tissue Collection, Processing, and Microscopic Analyses

The owner of the proband elected to have the dog euthanized at 6 years 8.5 months of age due to the progression of neurological signs. Following euthanasia, brain, eye, small intestine, and heart tissues were collected for microscopic analyses [42]. The tissues were preserved in “Immuno” fixative (cacodylate-buffered 3.5% paraformaldehyde and 0.05% glutaraldehyde) and fixative for electron microscopy (EM-fix) (cacodylate-buffered 2.0% glutaraldehyde, 1.12% formaldehyde) and prepared for fluorescence, light and electron microscopic evaluations as described previously [5]. 

For assessment of tissue autofluorescence, slices of the immuno-fixed tissues were embedded in an OCT medium (Tissue-Tek, Sakura Finetek, Torrance, CA, USA). The fixed specimens were incubated sequentially in 170 mM sodium cacodylate, pH 7.4, 10% sucrose in the cacodylate buffer, 20% sucrose in the cacodylate buffer and 1:1 20% sucrose:Tissue-Tek for a minimum of 45 min each. The samples were then incubated in Tissue-Tek in embedding molds for 45 min and then frozen on dry ice blocks. The frozen samples were cryo-sectioned at a thickness of 8 μm. The unstained sections were mounted on SuperFrost slides (Thermo Fisher Scientific, Waltham, MA, USA) in 170 mM sodium cacodylate, pH 7.4, and examined for autofluorescence using a Zeiss Axiophot microscope equipped with a Prior Lumen 200 light source, a 395–440 nm bandpass excitation filter, an FT 460 dichromatic beam splitter, and an LP 470 barrier filter. A 515 nm barrier filter was also placed in the emission light path. Fluorescence images were acquired using a Zeiss Neofluor 40× objective with a numerical aperture of 0.75 and an Olympus DP72 digital camera. Fluorescence microscopic evaluations of the retina were performed on sections obtained from along the superior–inferior midline. Slices of the cerebellar cortex and parietal lobe cerebral cortex of the brain, cervical spinal cord, the heart ventricle wall, and a small intestine cross section were also examined with fluorescence microscopy.

Slices of the EM-fixed tissues were paraffin-embedded, and sections were stained with periodic acid–Schiff reagent using standard histopathology techniques [43]. EM-fixed cerebellar cortex was also processed for electron microscopy [44]. This included secondary fixation in osmium tetroxide and embedding in epoxy resin. Sections of selected areas from each tissue were cut at thicknesses of 80 to 90 nm, mounted on copper grids, and stained with lead citrate and uranyl acetate. The sections were examined with a JEOL 1400 transmission electron microscope (Tokyo, Japan) equipped with Gatan digital camera (Pleasanton, CA, USA).

### 2.5. Molecular Genetic Analyses

Genomic DNA from the proband was prepared from EDTA-anticoagulated blood as previously described [36]. The DNA was submitted to the University of Missouri Genomics Technology Core Facility for library preparation and 2 × 150 bp paired-end sequencing on an Illumina NovaSeq 6000 sequencer. A previously described data-processing pipeline was used to align the sequence reads to a current canine reference genome assembly (Dog10K_Boxer_Tasha) and to analyze them in conjunction with reads from 450 other canine whole genome sequences previously generated by the University of Missouri Canine Genetics Laboratory that were used as unaffected controls. The control sequences were generated from both healthy dogs and dogs with a variety of hereditary disorders, including dogs with various forms of NCL. The whole genome sequences from the control cohort have been deposited in the NCBI Sequence Read Archive (SRA) (https://trace.ncbi.nlm.nih.gov/Traces/sra/sra.cgi, accessed on 1 April 2026) [5]. The whole genome sequence from the proband was also deposited in the SRA. The BioSample ID for the proband is SAMN55198325.

## 3. Results

### 3.1. Neurologic Phenotype

The dog was previously diagnosed at 4 years and 8 months of age with an extrahepatic portosystemic shunt, which was repaired with an ameroid constrictor. Neurologic signs prior to the shunt repair included circling and head pressing. The dog recovered well and was neurologically normal after surgery. At 5 years and 4 months of age and 9 months after surgery, the dog began showing different neurological signs, which included ataxia in all limbs, disorientation and visual deficits. Signs of visual deficits included falling off steps, misjudging distances and bumping into objects. As signs progressed, the owner also noted the dog showing other behavior changes such as aggression towards other dogs, urination accidents in the house, aimless circling, excessive whining and being easily startled.

Physical examination was normal with a 4 out of 9 body condition score. Orthopedic examination revealed bilateral carpal valgus. Recurrence of signs of hepatic encephalopathy was ruled out based on normal ammonia levels and pre- and post-bile acids testing. No significant abnormalities were identified on complete blood count (CBC), serum biochemistry analysis and urinalysis.

A complete neurological examination was performed. Mentation was quiet, dull and disoriented with resistance to restraint. Posture was kyphotic with a wide-based stance. Gait evaluation showed mild hypermetria with a general proprioceptive ataxia. A mild titubation of the head and body were observed at rest. The dog fell forward after neck extension and head drop. Cranial nerve evaluation revealed intact menace response with normal direct and indirect pupillary light reflexes. The oculocephalic reflex was delayed when moving the head in lateral and vertical directions. Ventrolateral strabismus was induced on neck extension. Facial sensory testing and reflexes were intact. Paw replacement was normal but thoracic limbs showed delayed movement on the wheelbarrow test and pelvic limbs were delayed in placement on extensor postural thrust. All spinal reflexes were intact. Neuroanatomic localization was diffuse involving the forebrain, cerebellum and central vestibular system. Neurodegenerative, inflammatory or neoplastic diseases were considered as differentials.

**Figure 1:**
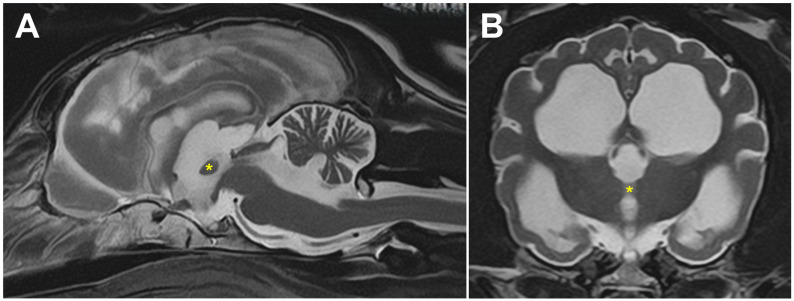
T2-weighted brain MRI in the sagittal (**A**) and transverse (**B**) planes at level of interthalamic adhesion from the affected dog. Diffuse brain atrophy is indicated by increased CSF surrounding the folia of the cerebellum, enlargement of the lateral, third and fourth ventricles, widening of the sulci of the cerebral cortex and decreased thickness of the interthalamic adhesion (asterisks).

MRI findings identified marked brain atrophy with dilation of the entire ventricular system (Figure 1). T2W, FLAIR and T1W sequences indicate typical fluid consistency of CSF. The brain atrophy was characterized by widening of the sulci in the cerebrum and cerebellar hemispheres, decrease in gray and white matter demarcation, thinning of corpus callosum, and decreased size of the thalamus and interthalamic adhesion. The pons and medulla were considered spared by showing no atrophy. No abnormal contrast enhancement or intracranial mass effects were detected. A cerebrospinal fluid analysis was not performed.

Based on the neurological signs and brain MRI, neuronal ceroid lipofuscinosis or another neurodegenerative disease was suspected. EDTA anti-coagulated blood from the dog was submitted to the University of Missouri Canine Genetics Laboratory for molecular genetic analysis.

#### Outcome

At 6 years and 8 months of age, the dog became more aggressive to the owner, developed complete loss of housebreaking skills, and had more difficulty eating and drinking without assistance. Changes from the initial neurological examination abnormalities included exaggerated responses to external stimuli, reluctance to ambulate with frequent falling and leaning, and resting tremors of head and trunk, which worsened with stress and movement. Intermittent myoclonic jerks were observed. Changes in the cranial nerve evaluation revealed absent menace response OU with sluggish and incomplete pupillary light reflexes. Paw replacement and hopping were delayed in all limbs. Due to the dog’s behavior changes and declining neurological status, the owner elected for humane euthanasia and consented to necropsy.

### 3.2. Ophthalmic Phenotype

**Figure 2:**
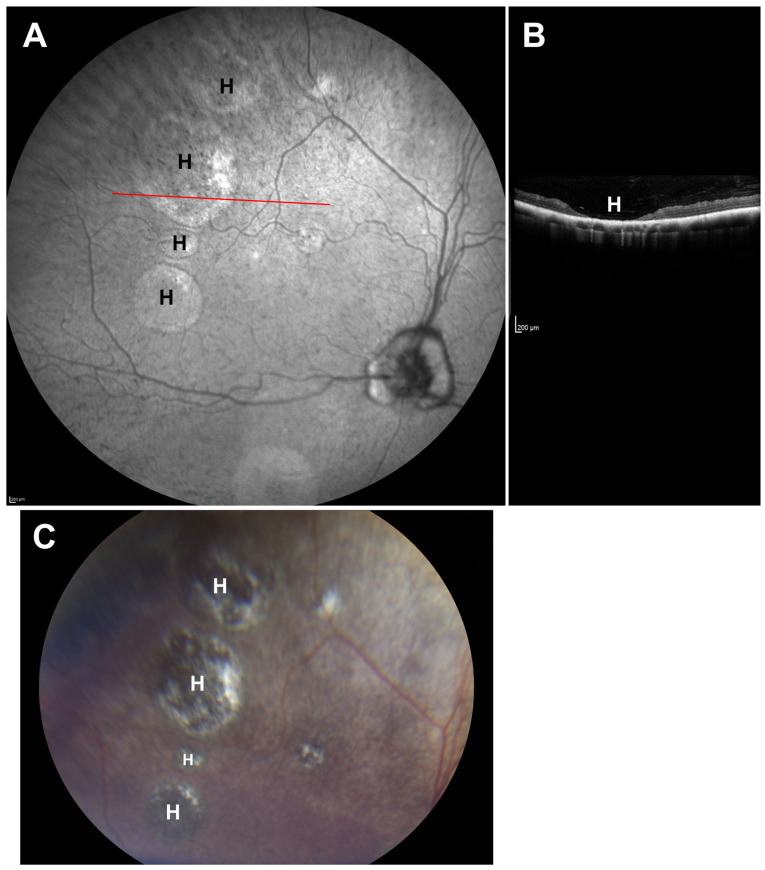
SLO (**A**) and fundus photo (**C**) images of the proband’s retina showing a series of holes in the retina (H). An OCT image of a scan taken at the position of the red line in (**A**) is shown in (**B**). H in this image indicates an area where the retina is thinned to complete absence, flanked by areas where the retina remains present.

**Figure 3:**
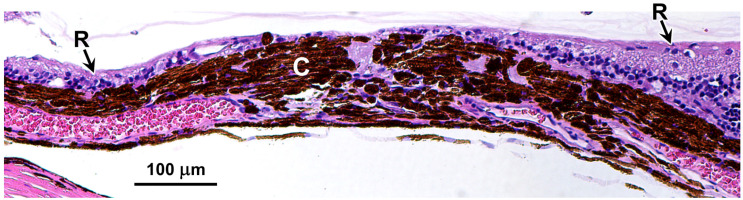
H&E-stained section of a cross-section of an area of the proband’s retina showing one of the retinal holes. The retina (R) tapers down at the edges of the hole leaving a bare choroid (C) at the center of the hole.

The owners reported observing progressive impairment in visually mediated behavior under both dim and bright light conditions starting at approximately 4.5 years of age. This was characterized by bumping into obstacles and misjudging distances. No abnormality was noted on Schirmer tear test-1, fluorescein stain and tonometry. Slit-lamp biomicroscopic examination revealed incipient anterior cortical cataracts in both eyes. Funduscopic evaluation demonstrated multifocal, irregularly shaped circular-to-ovoid regions of retinal atrophy, measuring approximately one-half to two-disk diameters (Figure 2A,C). These lesions were localized to the temporal fundus of the right eye and the nasal fundus of the left eye. The lesions were characterized by areas of dense hyperpigmentation interspersed with hypopigmented regions and white, lace-like patterns, accompanied by generalized mild attenuation of the retinal vasculature. OCT retinal imaging revealed severe retinal attenuation within the lesions (Figure 2B). Histological examination demonstrated that the retina was thinned to complete absence at the centers of the lesions (Figure 3).

### 3.3. Storage Material

**Figure 4:**
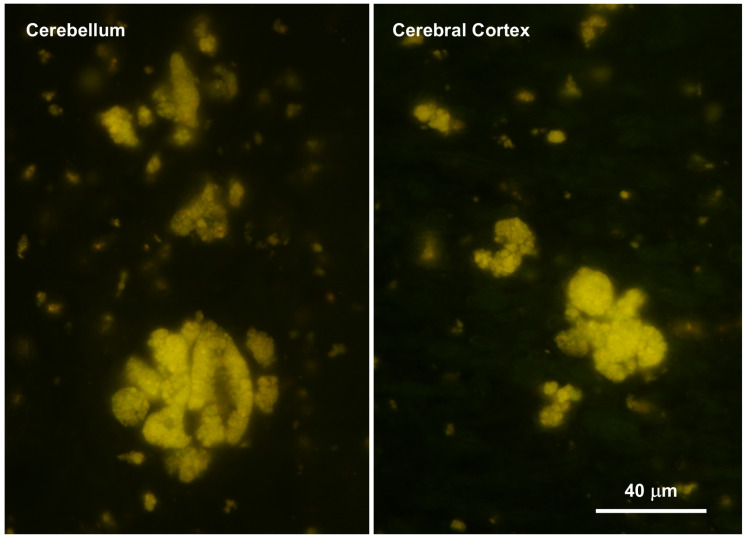
Fluorescence micrographs of unstained cryostat sections of cerebellar cortex and cerebral cortex from the proband. In the cerebellum large aggregates of autofluorescent inclusions were abundant in the granule cell layer but were not present in the Purkinje cells and were sparse in the molecular layer. Bar indicates magnification of both micrographs.

**Figure 5:**
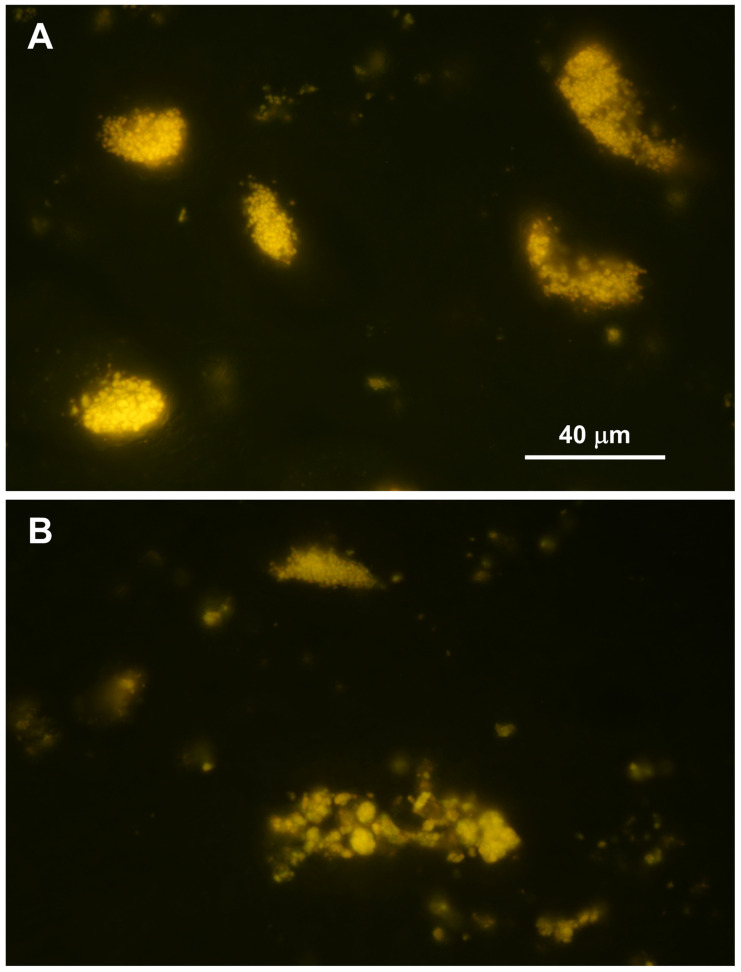
Fluorescence micrographs of unstained cryostat section of cervical spinal cord ventral horn (**A**) and white matter (**B**) from the proband. The motor neuron cell bodies contained large aggregates of autofluorescent inclusions (**A**). Similar aggregates of inclusions were present throughout the rest of the spinal cord (**B**). Bar in (**A**) indicates magnification of both micrographs.

**Figure 6:**
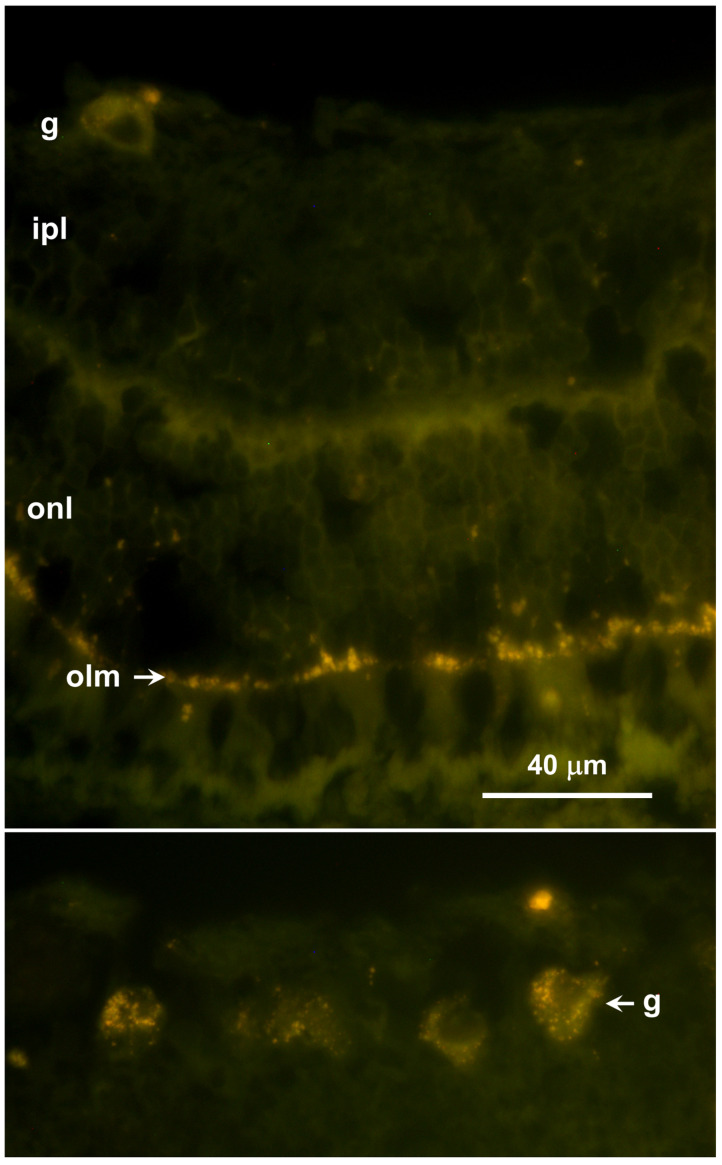
Fluorescence micrographs of unstained cryostat sections of mid-peripheral (**top**) and central (**bottom**) regions of the retina from the proband. Autofluorescent inclusions were present along the outer limiting membrane (olm) throughout the retina, and in ganglion cell bodies (g) in the central retina, but not in other layers of the retina (ipl: inner plexiform layer; onl: outer nuclear layer). Bar indicates magnification of both micrographs.

**Figure 7:**
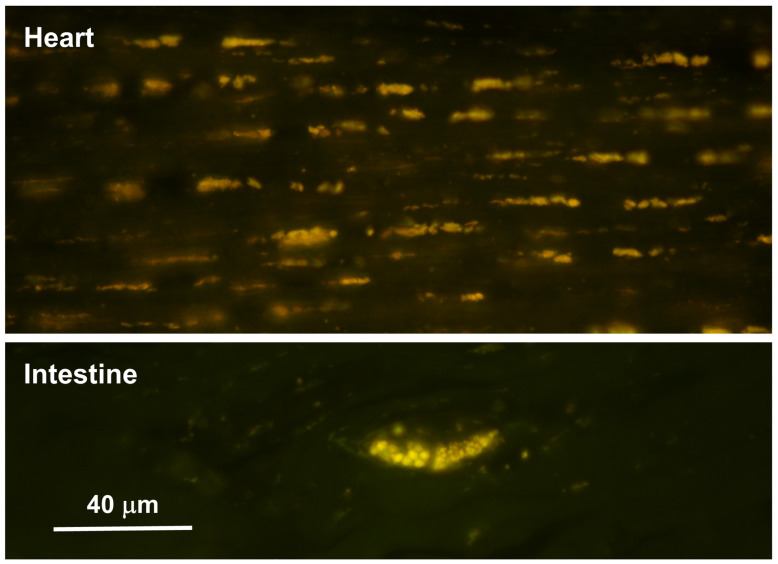
Fluorescence micrographs of unstained cryostat sections of cardiac ventricular wall muscle (**top**) and the wall of the small intestine from the proband (**bottom**). In the heart, autofluorescent inclusions were present in almost all muscle fibers. In the intestinal wall the inclusions were abundant in scattered cells. Bar indicates magnification of both micrographs.

Abundant autofluorescent inclusions were present in unstained sections of the cerebellar cortex, cerebral cortex, and spinal cord of the proband (Figure 4 and Figure 5). In the cerebellar cortex, the inclusions were present primarily in the granule cell layer and between the Purkinje cells, with minimal autofluorescent material observed within the Purkinje cells themselves, and relatively few in the molecular layer. In the cerebral cortex, the storage material was abundant in cells throughout all layers of the gray matter. In the spinal cord, the motor neurons contained large aggregates of autofluorescent inclusions (Figure 5A). Similar inclusions were present throughout the rest of the spinal cord, including in the white matter tracts (Figure 5B). In the retina, autofluorescent inclusions were present along the outer limiting membrane throughout the retina and in the cell bodies of ganglion cells near the optic nerve head (Figure 6). The amount of storage material in the ganglion cells was relatively modest. Abundant autofluorescent inclusions were also present in the heart ventricular muscle fibers (Figure 7) and in scattered cells in the wall of the small intestine (Figure 7).

**Figure 8:**
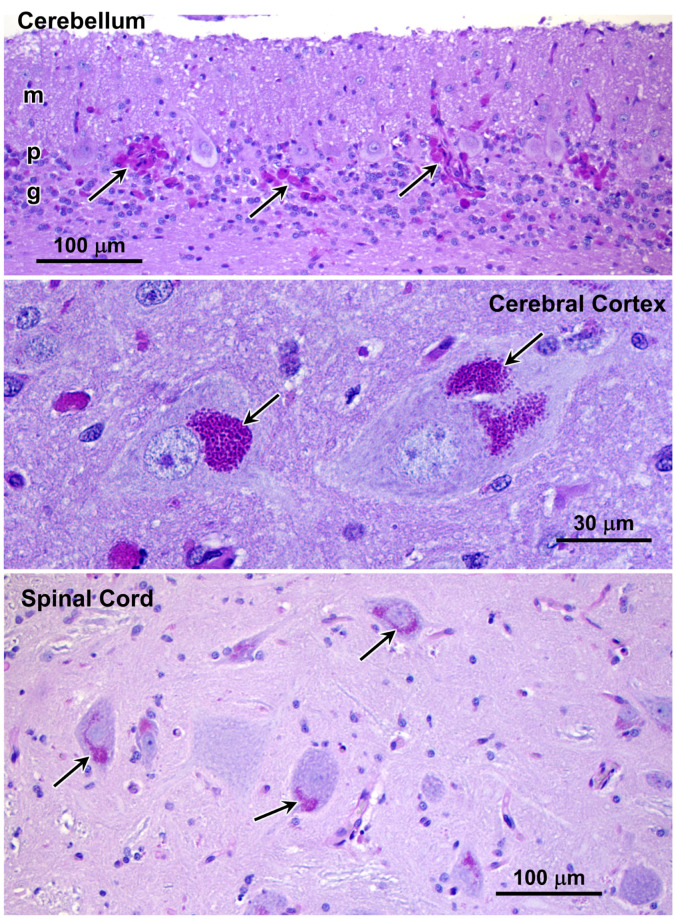
Light micrographs of paraffin sections of tissues from proband showing PAS-stained inclusions (arrows). In the cerebellum, the molecular (m), Purkinje cell (p), and granule cell (g) layers are labeled.

H&E- and PAS-stained paraffin sections of the cerebellum, cerebral cortex, and spinal cord were examined for pathological abnormalities. The folia of the cerebellar cortex were slender and separated by wider than normal sulci. The molecular layer was significantly thinner than normal. The granule cell layer was also quite thin with marked cellular depletion (Figure 8). Cytoplasmic inclusion bodies in cells located primarily in the granular layer stained with PAS (Figure 8). Almost no PAS-positive cytoplasmic inclusions were present in the Purkinje cells. In the cerebrum, there are scattered infiltrates of macrophages with occasional perivascular aggregates. Most neuronal cells contained PAS-positive cytoplasmic inclusion bodies (Figure 8). In the spinal cord, most neurons contained peri-nuclear aggregates of PAS-positive inclusions (Figure 8).

**Figure 9:**
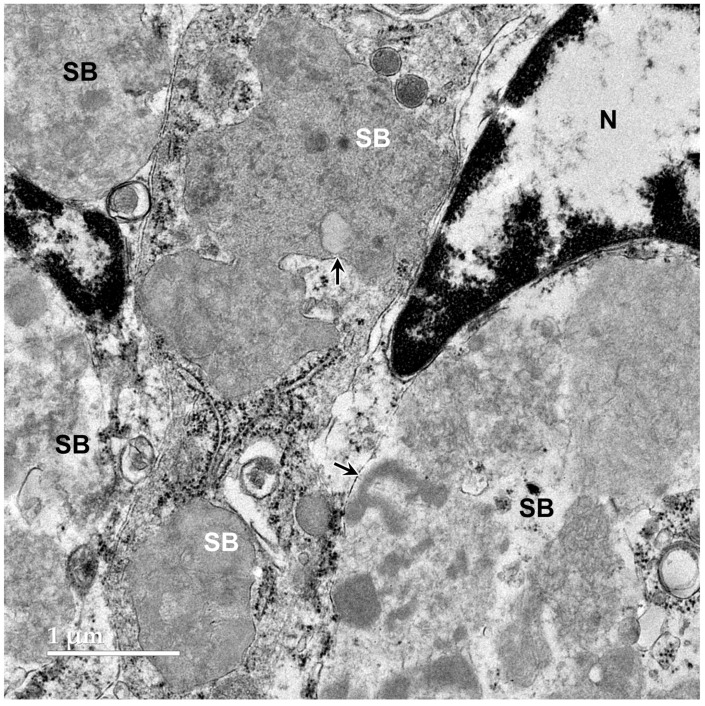
Electron micrograph of cells in the cerebellar cortex granule cell layer of the proband showing storage bodies (SBs) that varied in shape, size, and the appearance of their contents. The storage bodies were enclosed in single membranes (arrows). N: cell nucleus.

**Figure 10:**
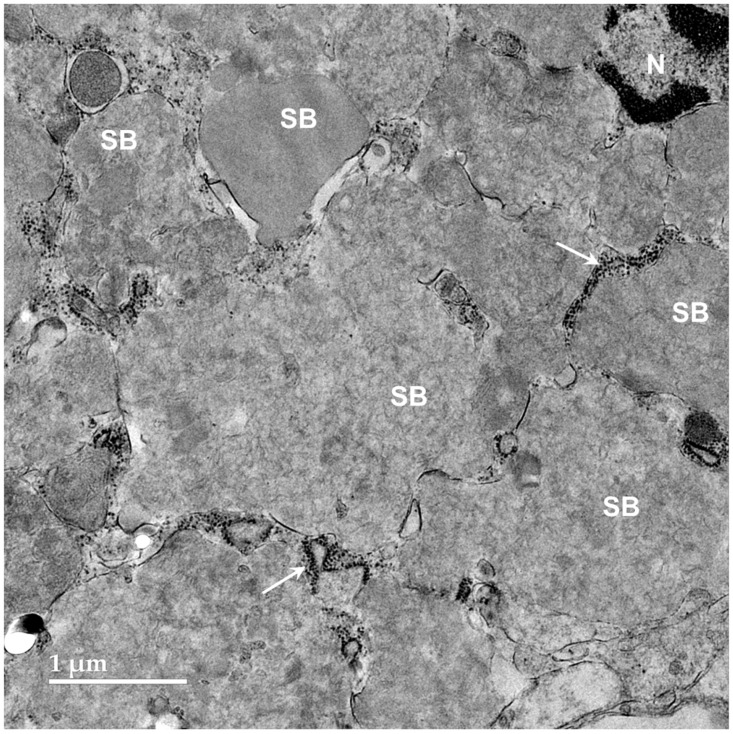
Electron micrograph of a tightly packed cluster of storage bodies (SBs) in a cerebellar cortex granule cell layer neuron. The storage bodies almost completely fill the cell body compressing the nucleus (N). Clusters of ribosomes were present in narrow gaps between some of the storage bodies (arrows).

Transmission electron microscopic examination of the cerebellar cortex granule cell layer of the proband showed the presence of numerous large storage bodies, some of which were more than 10 μm in diameter (Figure 9 and Figure 10). The storage bodies were variable in shape and were each surrounded by a single membrane. The storage body contents were heterogeneous in appearance, even among storage bodies in the same cell. A large proportion of the storage body contents consisted of membrane-like rectilinear and tubular structures (Figure 9 and Figure 10). In many cells, the perinuclear cell bodies were filled with tightly packed heterogenous storage bodies (Figure 10).

### 3.4. Molecular Genetics

The proband was genotyped for variants in *TPP1* and *PPT1* that were previously associated with forms of NCL in Dachshunds [41,45]. The dog did not have either of these variants. The whole genome sequence data of the proband were analyzed in conjunction with similar data generated with DNA from 395 dogs of numerous breeds that included healthy dogs and dogs with a variety of diseases. Among these were 11 Dachshunds. None of the dogs in this cohort had exhibited signs identical to those of the proband. These 395 whole genome sequences served as controls and allowed us to screen for variant alleles that were homozygous in the proband and rare or absent in the control population. We hypothesized that the disease was inherited as an autosomal recessive trait, although we were unable to obtain health information on the dog’s parents or littermates. The whole genome sequence of the proband was compared to the control sequences to identify variants that were uniquely homozygous in the proband.

Based on a recessive model of inheritance, variants identified with WGS analysis were filtered through a series of sequential steps that are summarized in Table 1. Relative to the Dog10K_Boxer_Tasha reference genome, the proband was found to be homozygous for 21,003 variants (since the proband was a male, variants on the X chromosome were called homozygous). The clinical signs exhibited by the proband were distinct from those of any other dog in the control cohort. Therefore, of the 21,003 variants, only 107 that were homozygous exclusively in the proband were considered as potentially causal candidates. To further narrow down the potential causal variants, any that were heterozygous in more than two dogs in the control cohort were excluded, leaving 35 variants for further consideration. Of these variants, 16 were predicted to alter the amino acid sequences of the encoded proteins and the remainder occurred in the 3′ and 5′ untranslated regions. These 16 candidate variants are listed in Table 2.

**Figure 11:**
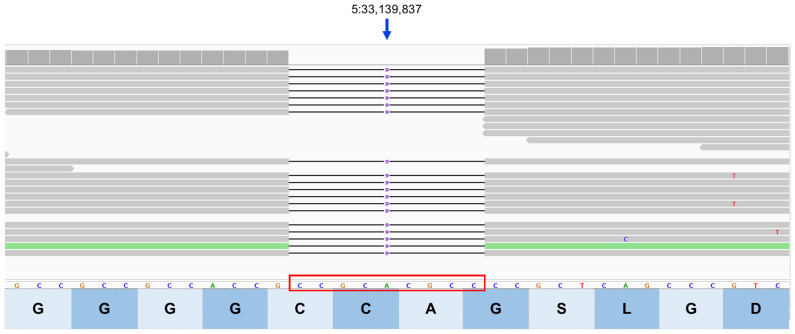
Integrative Genomics Viewer image of the proband’s whole genome sequence reads aligned to the reference sequence in the vicinity of position 33,139,837 on chromosome 5. The variant deletion is outlined in red. The reference DNA sequence is shown along with the predicted BORCS6 protein amino acid sequence. The nucleotide sequence change predicts a deletion of 3 amino acids (GAC; glycine, alanine, cysteine).

**Figure 12:**
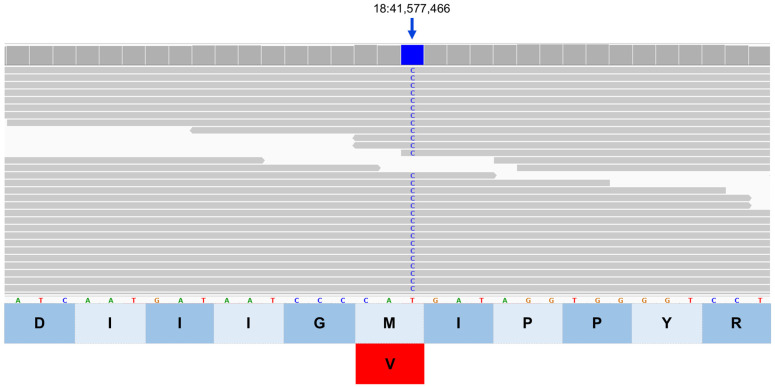
Integrative Genomics Viewer image of the proband’s whole genome sequence reads aligned to the reference sequence in the vicinity of position 41,577,466 on chromosome 18 (blue bar at top). The variant C is highlighted in blue. The reference DNA sequence is shown along with the predicted ATG13 protein amino acid sequence. The nucleotide sequence change predicts an amino acid change from methionine (M) to valine (V).

**Figure 13:**
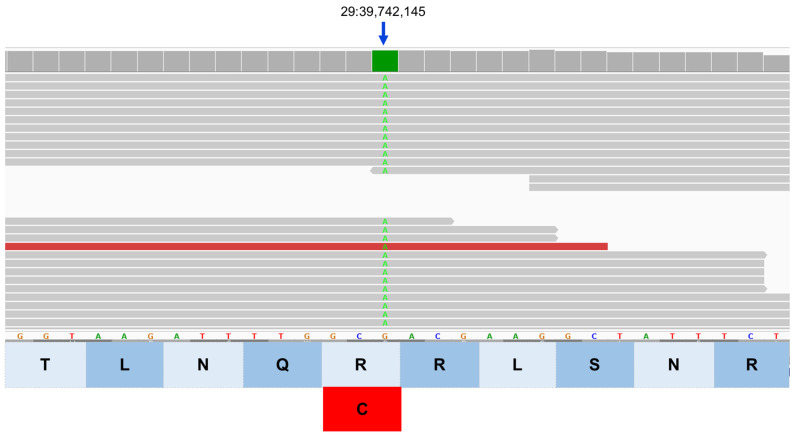
Integrative Genomics Viewer image of the proband’s whole genome sequence reads aligned to the reference sequence in the vicinity of position 39,742,145 on chromosome 29 (green bar at top). The variant A is highlighted in green. The reference DNA sequence is shown along with the predicted TP53INP1 protein amino acid sequence. The nucleotide sequence change predicts an amino acid change from arginine (R) to cysteine (C).

**Figure 14:**
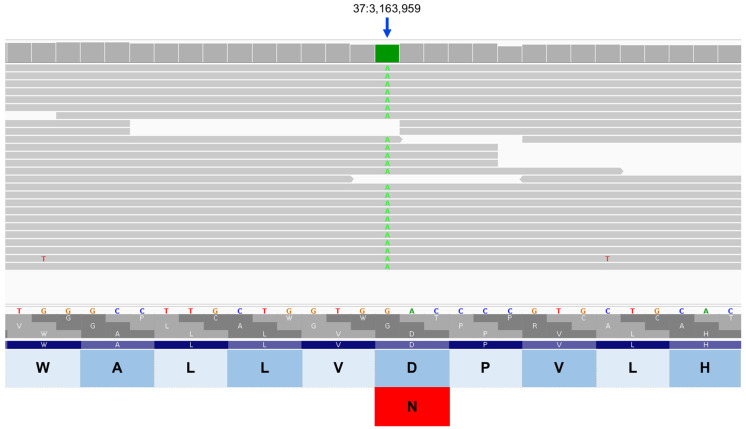
Integrative Genomics Viewer image of the proband’s whole genome sequence reads aligned to the reference sequence in the vicinity of position 3,163,959 on chromosome 37 (green bar at top). The variant A is highlighted in green. The reference DNA sequence is shown along with the predicted CLN8 protein amino acid sequence. The nucleotide sequence change predicts an amino acid change from aspartic acid (D) to asparagine (N).

**Figure 15:**
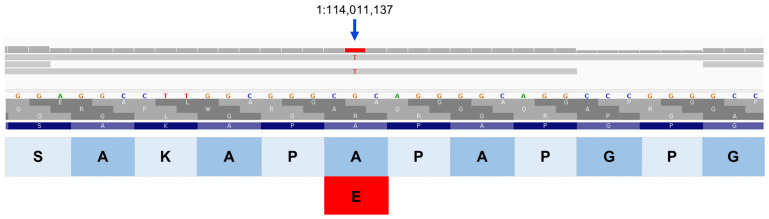
Integrative Genomics Viewer image of the proband’s whole genome sequence reads aligned to the reference sequence in the vicinity of position 114,011,137 on chromosome 1 (red bar at top). The variant T is highlighted in red. The reference DNA sequence is shown along with the predicted SPRED3 protein amino acid sequence. The nucleotide sequence change predicts an amino acid change from alanine (A) to glutamic acid (E).

Based on reviews of published information on the functions of the proteins encoded by these 16 genes, the variants in *SPRED3*, *TP53INP1*, *ATG13*, *CLN8*, and *BORCS6* appeared most likely to be associated with the disease phenotype of the proband because each of these genes encodes a protein involved in lysosomal degradation pathways [1,46,47,48,49,50,51,52,53,54,55,56,57,58,59,60,61,62,63,64]. The genotypes at these loci were confirmed by examining the WGS data for these regions with the Integrative Genomics Viewer (Figure 11, Figure 12, Figure 13, Figure 14 and Figure 15). The sequence variant call for *SPRED3* was made with low confidence because there was only 2× coverage at the variant site. Therefore, this variant was confirmed by Sanger sequencing of the proband’s DNA for this region of the gene. The amino acids at the equivalent positions across diverse mammalian species matched those in the canine reference sequence (Supplementary Figures S1–S5), suggesting that the amino acids at these positions have highly conserved functional roles.

Of the dogs in our WGS cohort, the proband was the only dog that had the *SPRED3* and *TP53INP* variant alleles. There was one dog in the control cohort that was heterozygous for the *ATG13* variant, a different dog that was heterozygous for the *BORCS6P* variant, and a third dog that was heterozygous for both the *CLN8* and *BORC96* variants (Table 3). All three of these were Dachshunds that did not exhibit any of the neurological signs that characterized the disorder in the proband. Our control cohort of 395 dogs included eight additional Dachshunds. None of these dogs had any of the variant alleles in the candidate genes (Table 3).

## 4. Discussion

The proband exhibited neurological signs similar to those of other dogs with various forms of NCL [24]. Most of the previously characterized canine NCLs have much earlier onset of signs and progression to end-stage disease. This includes two forms of NCL previously identified in Dachshunds associated with variants in *PPT1* and *TPP1* [41,45]. Although cases of adult-onset NCL-like disorders similar to that of the proband have also been reported in Dachshunds, the molecular genetic basis of the disorders was not determined for these dogs [65,66,67]. Other canine NCLs associated with variants in *ATP13A2* and *ARSG* have even later onsets than the disorder in the proband [7,34]. This mirrors the human NCLs in which ages of onset range from early infancy to adulthood, even among individuals with different variants in the same gene [3]. Based on the clinical signs, brain and retinal atrophy, and the intracellular accumulation of autofluorescent storage material, it is consistent with established practice to classify the disorder in the proband as an NCL. In addition, the ultrastructure of the storage body contents was similar to that of some forms of canine as well as human NCLs [1,36,40,41,68], although the storage bodies in the proband’s cerebellar cortex and cerebral cortex were larger and more densely packed and irregular in shape than in most other NCLs. The distribution of storage material within the cerebellar cortex was also unique among the reported canine NCLs. A comparison of the major phenotypic features of the disorder with those of other canine NCLs is summarized in Table 4.

Perhaps the most unique feature of the disease in the proband was the formation of large focal areas of complete retinal atrophy. Other forms of canine NCL are also characterized by progressive visual impairment, but this is usually accompanied by uniform degeneration across the entire retina. An exception is CLN2 disease in Dachshunds with a null variant in *TPP1*. Some, but not all, dogs with this disorder develop focal retinal detachments [69]. As the disease progresses, the detachments resolve, but the retina in these areas is significantly thinned, although it does not degenerate completely as it did in the affected areas of the proband’s retina. In Dachshund CLN2 disease, the onset of clinical signs occurs at about 4 months of age and progresses to end stage and death by 12 months of age. It is possible that the retinal degeneration in the affected areas would have further progressed had the dogs survived longer. The retinas of the proband in the current study were not examined until the disease was in an advanced stage, so it is not known whether the focal areas of degeneration were detached from the underlying retinal pigment epithelium and choroid earlier in the disease progression. It is possible that the mechanisms underlying focal retinal pathology were similar in the two forms of NCL, and that the differences were due mainly to differences in rates of disease progression. It should be possible to investigate this possibility if dogs with the same disorder as the proband can be identified and examined earlier in the disease progression.

Most of the human and canine NCLs have been associated with DNA sequence variants in a single gene. Among the variants for which the proband was the only homozygote in our WGS cohort was a missense variant in *CLN8* that predicted a D88N change in the protein amino acid sequence. Other *CLN8* variants underlie several other canine NCLs, as well as human NCLs [2,3,35,36,37,38,39]. Thus, it appears possible that this variant alone is responsible for the disorder in the proband. However, the specific *CLN8* D88N variant in the proband has not been reported to be associated with any human or canine NCL cases. For other variants in this gene in dogs with NCL, the onset of clinical signs and end-stage disease occur much earlier [35,36,37,38,39]. In addition, the patterns of autofluorescent storage body accumulation and brain and retinal atrophy in the proband were different from those associated with *CLN8* variants. In particular, the massive accumulation of the storage material specifically in the granular layer of the cerebellum does not occur in NCL-affected dogs with *CLN8* variants. In addition, the unusual pattern of retinal degeneration has not been observed in any of the established animal or human NCLs associated with *CLN8* variants [2,3,35,36,37,38,39,70]. The selective thinning of the cerebellar molecular and granule cell layers is also unique among the canine NCLs, as was the presence of autofluorescent inclusions in the white matter tracts of the spinal cord. Thus, it is not possible to rule out contributions to disease pathogenesis from the other homozygous variants that were unique to the proband. Unfortunately, we were unable to obtain disease status information or DNA samples from any close relatives of the proband that may have enabled us to rule out contributions from these other variants. The owners of the proband provided us with contact information for the dog’s breeder, but the breeder did not respond to our inquiries about the proband’s littermates and parents.

Of the 16 variants for which the proband was the only homozygote among the dogs in our WGS cohort were those in four other genes, in addition to *CLN8*, that encode proteins involved in lysosomal pathways [51,52,53,54,55,56,57,58,59,60,61,62,63,64]. Most of the genes that have been associated with human and animal NCLs are also involved in various aspects of lysosomal function. Thus, it is just as plausible that any of these variants, either alone or in combination with each other, could be responsible for the disorder in this dog as it is that the *CLN8* variant alone causes the disorder. Indeed, a combination of variants in two genes that encode proteins involved in autophagy was associated with an NCL-like disorder in a Petit Bleu de Gascogne dog [5]. For any other Dachshund presenting with similar signs to those of the proband, all five of the candidate variants should be considered as potentially being involved in the disease pathogenesis. Based on the genotypes of the other 11 Dachshunds in our WGS cohort, each of these five variants is probably rare in the breed. However, two Dachshunds were heterozygous for the *BORCS6* variant, one Dachshund was heterozygous for the *CLN8* variant, and another for the *ATG13* variant. Therefore, screening a large number of Dachshunds for these variants may identify homozygotes and allow us to determine whether any of these variants alone may be responsible for the disorder.

The proband in this study had an unusual disease phenotype that has not been documented in other Dachshunds. Although the neurological signs overlap those of other canine NCLs, the retinal phenotype with scattered areas of complete retinal degeneration is unique among this group of diseases. Thus, it will be important to examine the retinas of any Dachshunds presenting with neurological signs similar to those of the proband. Identifying additional dogs with the same disorder and their relatives should enable us to further assess the potential involvement of the candidate genes in the disease. Other cases of adult-onset NCL in Dachshunds have been described, most recently in 2024, but the molecular genetic basis of the disorder has not been identified in any of these cases [65,66,67], and DNA samples from them are not available for analysis. If DNA samples can be obtained from future Dachshund cases exhibiting similar signs to those of the proband, it should be possible to better assess the potential roles of each of the candidate variants in the disease pathogenesis, particularly if samples can also be obtained from affected and unaffected littermates.

A limitation of this study was that only a single affected dog was identified and we were unable to obtain DNA samples and health information on the parents or littermates of the proband. Had we been able to perform WGS on such relatives, we may have been able to eliminate some of the candidate DNA sequence variants, particularly if another of these dogs suffered from the same disorder. Although this late-onset disease appears to be rare, other similar cases have been reported. If additional affected dogs can be identified, WGS analysis can be performed to determine whether they have some or all of the same variants identified in the proband.

## 5. Conclusions

A Dachshund presented with an adult-onset progressive neurodegenerative disorder with clinical signs and histopathological features consistent with classification of the disorder as a form of NCL. However, the pattern of autofluorescent storage material accumulation and the nature of the disease-related retinal and cerebellar pathology were distinct from previously characterized NCLs. Whole genome sequence analysis identified variants in five genes that were uniquely homozygous in the proband and that encoded proteins involved in lysosomal degradative pathways. The data suggest that the dog had a novel form of NCL that may be caused by the effects of a combination of these variants.

## Figures and Tables

**Table 1:** WGS variant sequential filtering steps.

Filtering Steps	Number of Variants
Proband was homozygous relative to the Dog10K_Boxer_Tasha reference	21,003
Proband was the only homozygote among 395 other dogs	107
Two or fewer heterozygotes in control cohort	35
Variant predicted to alter the amino acid sequence of encoded protein	16

**Table 2:** Candidate disease variants.

Chr.	Position ^1^	Ref ^2^	Alt ^3^	Effect	AA Change	Gene ID
1	82528139	G	C	Missense	G113A	*RFK*
1	114011137	G	T	Missense	A328E	*SPRED3*
1	87580840	GCGGGCG-GC	G	Frameshift	RGRR90	*KLF9*
5	64516257	G	A	Missense	R177H	*CBFA2T3*
5	33139837	GCCGCACGCC	G	Deletion	GACG165G	*BORCS6*
18	41577466	T	C	Missense	M208V	*ATG13*
20	57520816	C	A	Missense	A8S	*WDR18*
27	28157196	CCCCGCCT-CCCGCCT	CCCCGCCT	Frameshift	EAG165	*TMTC1*
29	5034736	C	T	Missense	A374T	*ENSCAFG00000053177*
29	39742145	G	A	Missense	R210C	*TP53INP1*
31	37993317	G	A	Missense	G674D	*ADARB1*
32	17989002	T	C	Missense	E785G	*NFKB1*
32	20497995	T	C	Missense	V81A	*ENSCAFG00000010410*
37	3163959	G	A	Missense	D88N	*CLN8*
X	69593247	G	GA	Frameshift	T69T?	*AMMECR1*
X	21989401	C	T	Missense	G890R	*BCOR*

^1^ Positions based on Dog10K_Boxer_Tasha genome assembly. ^2^ Reference allele. ^3^ Proband allele. Variants shaded in green occur in genes that encode proteins involved in lysosome function.

**Table 3:** Genotypes of Dachshunds in the WGS control cohort for each candidate variant.

		Heterozygous				
Dog ID	Submitted For	*ATG13*	*CLN8*	*BORCS6*	*SPRED3*	*TP53INP1*
111855	Polymicrogyria	No	No	No	No	No
103075	Ataxia	No	No	No	No	No
113889	Methemoglobinemia	No	No	No	No	No
123438	Ehlers-Danlos	No	No	No	No	No
126339	Metabolic Encephalopathy	Yes	No	No	No	No
133552	Ehlers-Danlos	No	No	No	No	No
127323	Ehlers-Danlos	No	No	Yes	No	No
126655	Ehlers-Danlos	No	No	No	No	No
123742	Ehlers-Danlos	No	No	No	No	No
124330	Ehlers-Danlos	No	Yes	Yes	No	No
147704	NCL	No	No	No	No	No

Highlight indicates heterozygosity.

**Table 4:** Comparison of disease phenotype in proband with other canine NCLs.

Disease Phenotypic Feature in Proband	Other Canine NCLs
Autofluorescent storage material accumulation (AFSM)	All forms
AFSM cerebellum	All forms
AFSM cerebral cortex	All forms
AFSM cerebellum mainly in granule cell layer	No other form
AFSM cerebellum large clusters	Only some other forms
AFSM cerebral cortex large clusters	Only some other forms
AFSM spinal cord motor neurons	Most other forms
AFSM white matter tracts	Only some other forms
AFSM retinal ganglion cells	Multiple other forms
AFSM retinal outer limiting membrane	Only some other forms
AFSM cardiac muscle	Most other forms
AFSM intestine wall	Not evaluated in other forms
Substantial thinning of cerebellum granule cell layer	Only some other forms
Storage material stains with PAS	Only some other forms
Storage material contents primarily curvilinear	Only some other forms
Onset of clinical signs after 5 years of age	Only some other forms
Diffuse brain atrophy	Most other forms
Focal areas of complete retinal atrophy	No other form
Impaired vision	Most other forms
Ataxia	All forms
Development of aggressive behaviors	Only some other forms
Incontinence	Only some other forms
Compulsive behaviors	Only some other forms
Resting tremors	Most other forms
Myoclonic jerks	Some other forms
Dull mentation	Most other forms
Absence of grand mal seizures	Multiple other forms

## Data Availability

The original data presented in the study are openly available in NCBI Sequence Read Archive at https://trace.ncbi.nlm.nih.gov/Traces/sra/sra.cgi (accessed on 1 April 2026).

## Supplementary Materials

The following supporting information can be downloaded at https://www.mdpi.com/article/10.3390/genes17040465/s1: Supplementary Figures showing multi-species amino acid sequence alignments for proteins encoded by the candidate genes. Figure S1. Partial multi-species amino acid sequence alignment for SPRED3. Figure S2. Partial multi-species amino acid sequence alignment for BORCS6. Figure S3. Partial multi-species amino acid sequence alignment for ATG13. Figure S4. Partial multi-species amino acid sequence alignment for TP53INP1. Figure S5. Partial multi-species amino acid sequence alignment for CLN8.

## Abbreviations

The following abbreviations are used in this manuscript:

NCL	Neuronal ceroid lipofuscinosis
WGS	Whole genome sequencing
NCBI	National Center for Biotechnology Information
SRA	Sequence Read Archive
