# Supplementary figures and images for: Neuronal Ceroid Lipofuscinosis-like Disorder in a Dachshund with Sequence Variants in Lysosome-Related Genes
Source: Genes (Basel). 2026 Apr 15;17(4):465. doi: 10.3390/genes17040465 (PMC13116516; doi:10.3390/genes17040465)

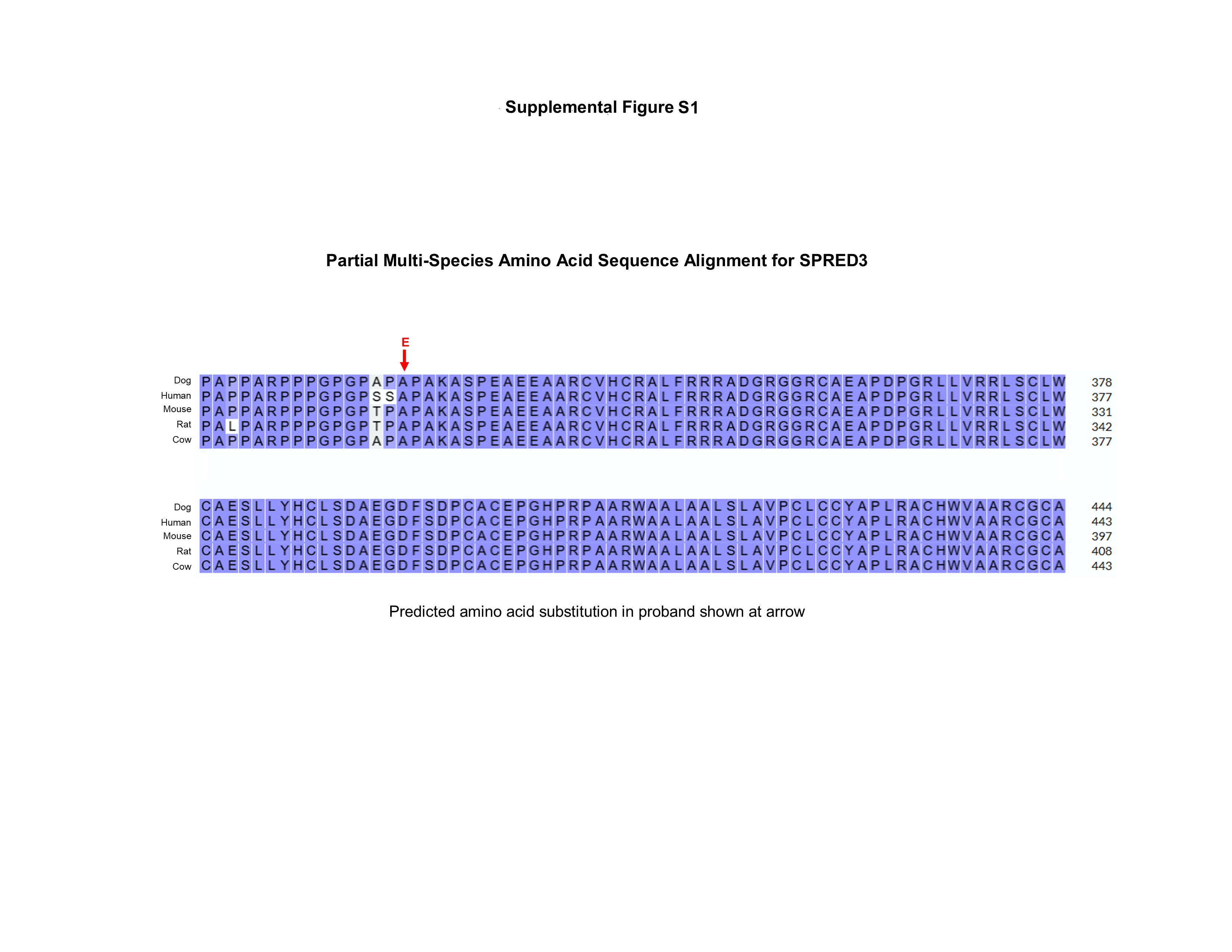

Supplement: Supplementary file 1 [file genes-17-00465-s001.zip › Sup Fig S1 SPRED3 AA Align.tif]

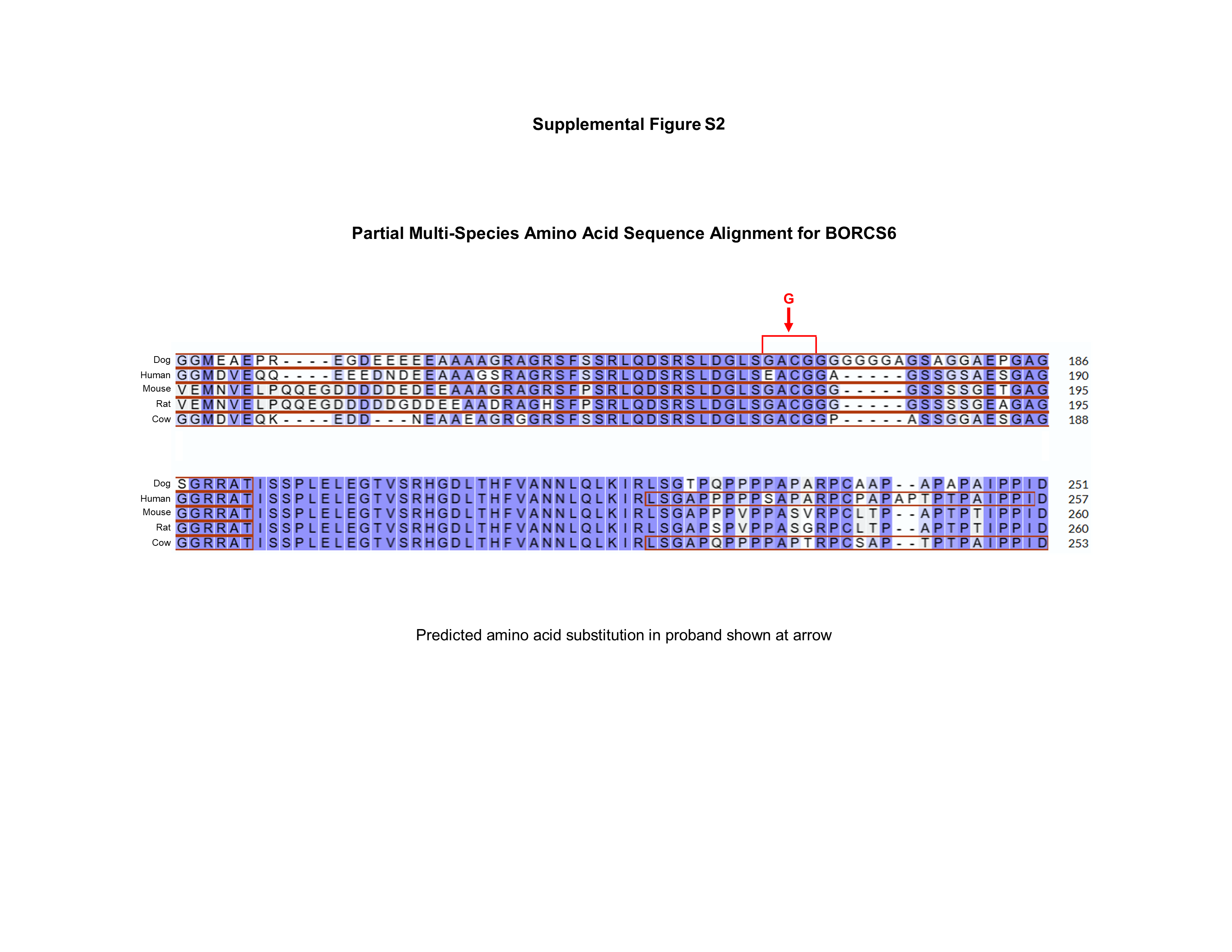

Supplement: Supplementary file 1 [file genes-17-00465-s001.zip › Sup Fig S2 BORCS6 AA Align.tif]

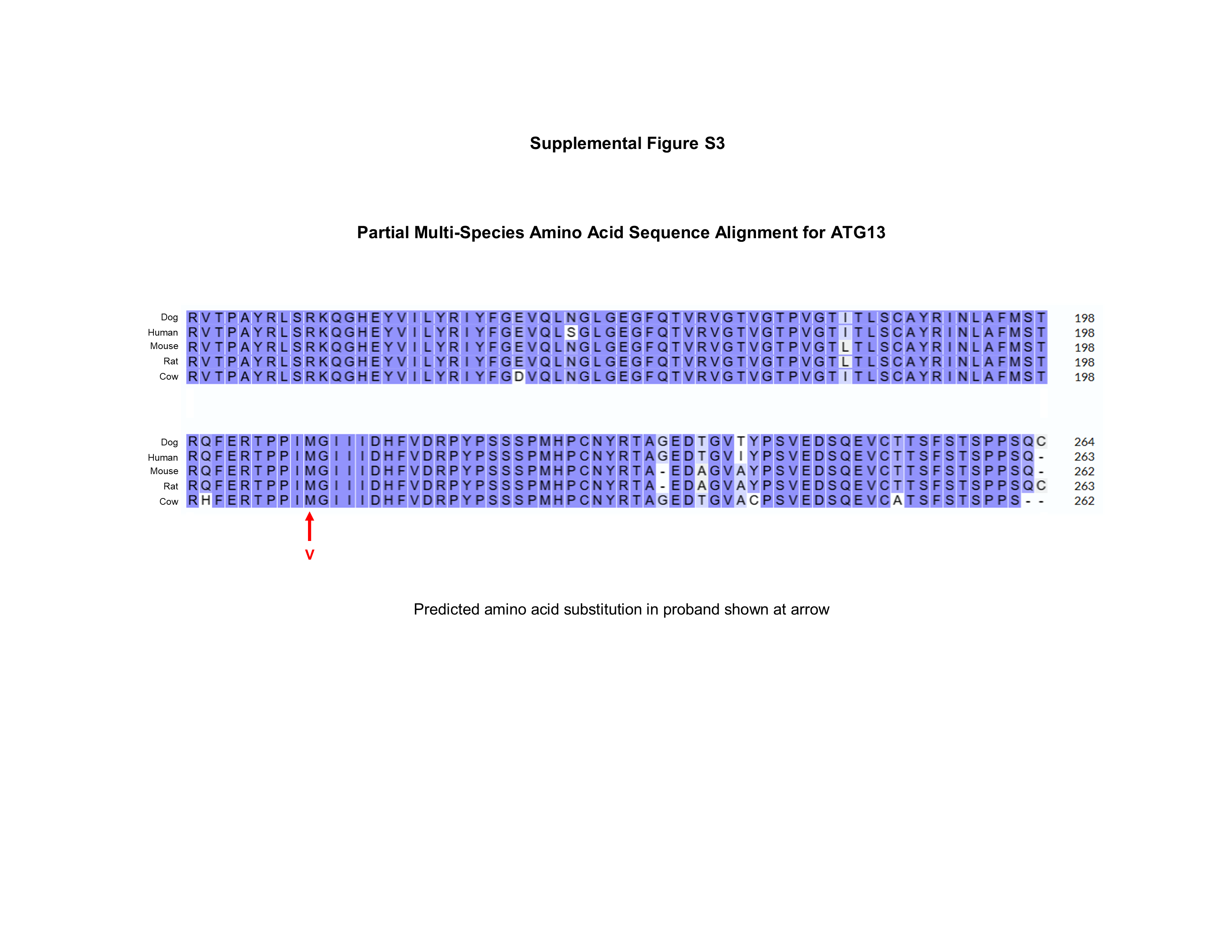

Supplement: Supplementary file 1 [file genes-17-00465-s001.zip › Sup Fig S3 ATG13 AA Align.tif]

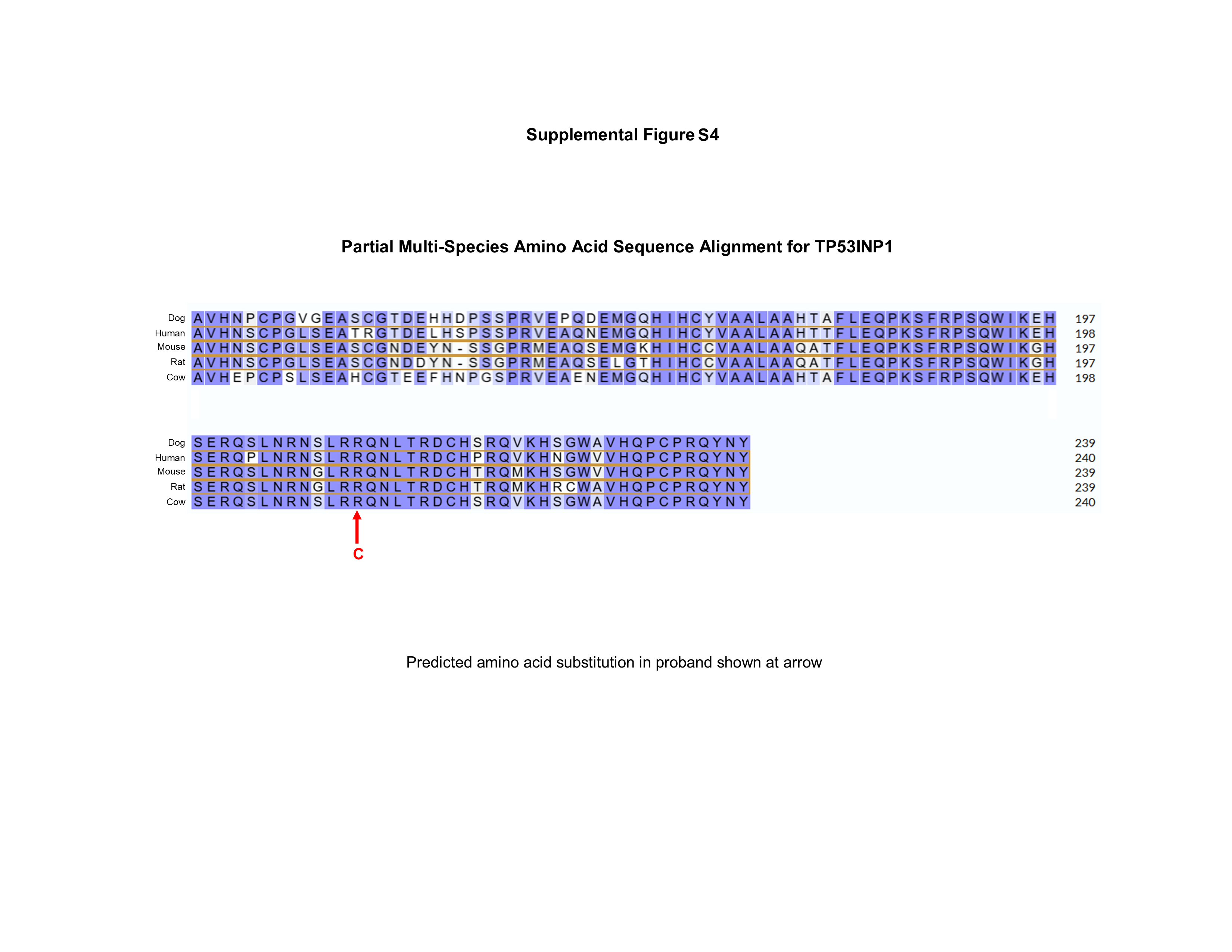

Supplement: Supplementary file 1 [file genes-17-00465-s001.zip › Sup Fig S4 TP53INP1 AA Align.tif]

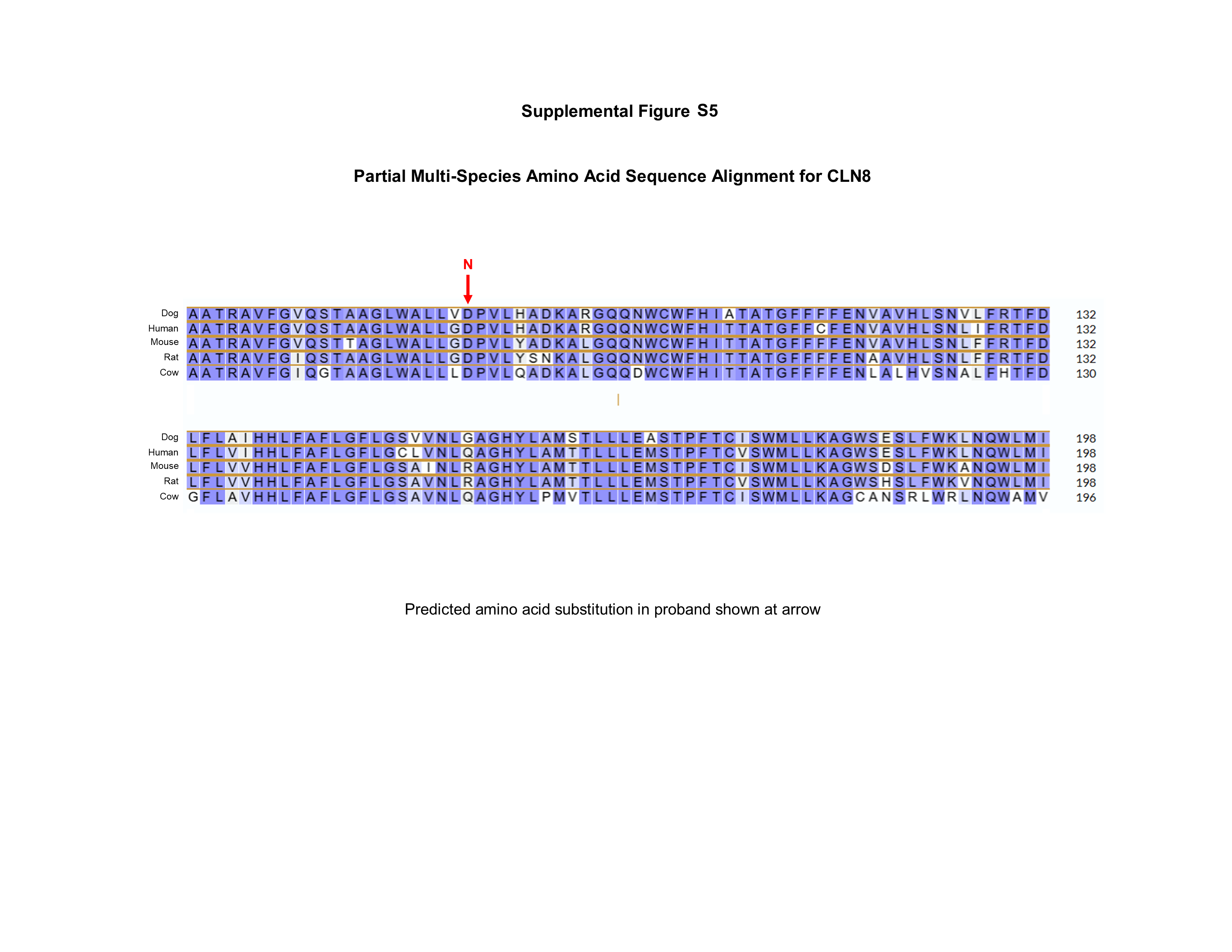

Supplement: Supplementary file 1 [file genes-17-00465-s001.zip › Sup Fig S5 CLN8 AA Align.tif]
